# Studying m^6^A in the brain: a perspective on current methods, challenges, and future directions

**DOI:** 10.3389/fnmol.2024.1393973

**Published:** 2024-04-22

**Authors:** Matthew Tegowski, Kate D. Meyer

**Affiliations:** ^1^Department of Biochemistry, Duke University School of Medicine, Durham, NC, United States; ^2^Department of Neurobiology, Duke University School of Medicine, Durham, NC, United States

**Keywords:** RNA, m^6^A, epitranscriptome, methods, brain

## Abstract

A major mechanism of post-transcriptional RNA regulation in cells is the addition of chemical modifications to RNA nucleosides, which contributes to nearly every aspect of the RNA life cycle. *N*^6^-methyladenosine (m^6^A) is a highly prevalent modification in cellular mRNAs and non-coding RNAs, and it plays important roles in the control of gene expression and cellular function. Within the brain, proper regulation of m^6^A is critical for neurodevelopment, learning and memory, and the response to injury, and m^6^A dysregulation has been implicated in a variety of neurological disorders. Thus, understanding m^6^A and how it is regulated in the brain is important for uncovering its roles in brain function and potentially identifying novel therapeutic pathways for human disease. Much of our knowledge of m^6^A has been driven by technical advances in the ability to map and quantify m^6^A sites. Here, we review current technologies for characterizing m^6^A and highlight emerging methods. We discuss the advantages and limitations of current tools as well as major challenges going forward, and we provide our perspective on how continued developments in this area can propel our understanding of m^6^A in the brain and its role in brain disease.

## Introduction

RNAs contain over 170 distinct chemical modifications which play important roles in regulating RNA processing and function. Although most of these modifications occur in non-coding RNAs such as ribosomal RNA and tRNA, recent studies have revealed a diverse and dynamic “epitranscriptome” within cellular mRNAs as well. *N*^6^-methyladenosine (m^6^A) is the most abundant internal mRNA modification and is found in thousands of cellular mRNAs, in addition to a large number of non-coding RNAs. m^6^A plays important roles in several RNA processing events, including splicing, nuclear export, stability, and translation, making it a critical regulator of gene expression in cells (Murakami and Jaffrey, 2022; Flamand et al., 2023). Indeed, m^6^A contributes to a wide variety of physiological processes, including development, innate immunity, gametogenesis, and the cellular stress response. Additionally, and consistent with its importance for cellular function, m^6^A dysregulation has been implicated in a variety of human diseases, including several cancers (Yang et al., 2020; He and He, 2023). Thus, understanding m^6^A distribution, regulation, and function is critical for advancing our knowledge of human health and disease.

Within the brain, m^6^A levels are particularly abundant compared to other tissues (Meyer et al., 2012; Liu et al., 2020), and proper regulation of m^6^A is critical for processes such as neural stem cell function, brain development, learning and memory, response to stress, and neuronal signaling (Flamand and Meyer, 2019; Livneh et al., 2020).

Our current knowledge of m^6^A has been accelerated by technological advances which have enabled the identification of m^6^A sites transcriptome-wide. Additionally, emerging technologies for targeted m^6^A manipulation in select RNAs are promising tools that can enable functional studies of m^6^A in the brain and other tissues. Here, we review m^6^A detection and manipulation strategies and discuss major challenges that need to be overcome. We also provide our perspective on future directions and areas that are likely to drive the field forward.

## m^6^A function and regulation in the brain

m^6^A is deposited in the nucleus co-transcriptionally by a large methyltransferase complex which includes METTL3 as the catalytic subunit and several additional accessory proteins including METTL14, WTAP, HAKAI, VIRMA, ZC3H13, and RBM15/15B (Shi et al., 2019; Zaccara et al., 2019; Flamand et al., 2023). Methylation occurs preferentially within the DRACH (D = A, G, U; R = A, G; H = A, C, U) consensus sequence, and recent studies have revealed that sequence specificity and gene architecture are the major determinants of methylation within cellular mRNAs (Garcia-Campos et al., 2019; Yang et al., 2022; He et al., 2023; Uzonyi et al., 2023). In addition, m^6^A can be removed by two eraser proteins, FTO and ALKBH5, which can contribute to dynamic regulation of m^6^A and gene expression under certain contexts (Shi et al., 2019; Flamand et al., 2023).

m^6^A has been shown to influence nearly every aspect of the RNA life cycle, including splicing, export, stability, localization, and translation (Shi et al., 2019; Zaccara et al., 2019; Flamand et al., 2023). However, the most well-established function of m^6^A in mRNAs is to recruit RNA degradation machinery through the binding of YTHDF proteins (Shi et al., 2019; Zaccara et al., 2019; Kontur et al., 2020; Zaccara and Jaffrey, 2020; Flamand et al., 2023). This m^6^A-dependent control of mRNA stability is critical for proper brain development, as this mechanism helps regulate the abundance of mRNAs that participate in neuronal stem cell function and cell cycle regulation (Yoon et al., 2017; Wang et al., 2018). m^6^A has also been shown to regulate mRNA metabolism in the brain in other ways, including promoting translation and nuclear export (Shi et al., 2019; Zaccara et al., 2019; Flamand et al., 2023). These functions are mediated by a variety of m^6^A reader proteins. For instance, YTHDF1 promotes methylated mRNA translation in neurons to control synaptic activity and learning and memory (Shi et al., 2018; Zou et al., 2023), and YTHDF2 promotes the differentiation of neural progenitors by degrading methylated transcripts (Li et al., 2018). The fragile X messenger ribonucleoprotein (FMRP) has been shown to preferentially bind methylated transcripts and facilitate their nuclear export (Edens et al., 2019). Additionally, our group identified RBM45 as a brain-enriched m^6^A reader protein that can impact splicing and regulate neuronal differentiation (Choi et al., 2022).

In addition to cortical development and neurogenesis, m^6^A also has important roles in regulating the function of mature neurons. Neurons are highly polarized cells, with complex dendritic processes that can make thousands of synaptic connections with other neurons. Proper synaptic function and plasticity requires the trafficking and local translation of mRNAs to synapses in an activity-dependent manner (Doyle and Kiebler, 2011; Holt et al., 2019; Roy et al., 2020). RNA localization is mediated by a variety of *cis*-acting elements, such as sequence and structure, which are bound by RNA-binding effector proteins (Doyle and Kiebler, 2011). m^6^A profiling of synaptic RNAs showed that several methylated transcripts are localized at synapses, suggesting that m^6^A could serve as an additional *cis*-acting element to control RNA localization in neurons (Merkurjev et al., 2018). Indeed, subsequent work from our group showed that hundreds of transcripts, including many that encode proteins important for synaptic maintenance and plasticity, are localized to distal processes in neurons in an m^6^A-dependent manner (Flamand and Meyer, 2022). We further showed that this is mediated through YTHDF proteins. However, why some methylated transcripts are degraded by YTHDF proteins while others are transported to distal processes is unknown, and it likely depends on other context-dependent factors, such as additional sequence and structural elements and interactions with other RBPs.

Interestingly, a recent study showed that mRNA stability is a major determinant of mRNA localization in neurons, with more stable transcripts being enriched in neurites (Loedige et al., 2023). The authors reported that neurite-enriched RNAs have lower levels of m^6^A, and they found that disrupting m^6^A or other factors that control RNA stability promotes neurite enrichment of neuronal transcripts. These studies examined m^6^A-mediated localization in primary cortical neurons, in contrast to hippocampal neurons used in our work, so it is possible that m^6^A may have unique roles in different neuronal subtypes. However, even within hippocampal neurons, we identified many transcripts with increased neurite localization following *Mettl3* depletion in addition to the hundreds of transcripts that showed decreased neurite localization (Flamand and Meyer, 2022). Thus, the effects of m^6^A on RNA localization may be transcript-specific. Further studies will be necessary for defining the cell type-and transcript-dependent effects of m^6^A on RNA localization in the brain.

In addition to RNA localization, recent work has demonstrated that m^6^A promotes local, activity-dependent translation of mRNAs in hippocampal neurons. This process is mediated by YTHDF1, which is required in hippocampal neurons for proper learning and memory (Shi et al., 2018). Supporting these data, deletion of *Mettl3* in the mouse hippocampus also leads to impaired learning and memory (Zhang et al., 2018). Furthermore, a mechanism by which YTHDF1 can promote activity-dependent translation in the hippocampus has been uncovered. Basal interactions between FMRP and YTHDF1 sequester YTHDF1. However, FMRP is phosphorylated upon neuronal activity, resulting the release of YTHDF1, allowing it to promote the translation of methylated transcripts (Zou et al., 2023). Altogether, m^6^A has been shown to regulate neuronal development and function by regulating RNA stability, localization, and translation.

## Current methods and recent advances in m^6^A mapping

The first method for transcriptome-wide m^6^A mapping was developed in 2012 and involved using m^6^A antibodies to immunoprecipitate methylated RNAs followed by next-generation sequencing to identify the methylated targets (Dominissini et al., 2012; Meyer et al., 2012). This method, called MeRIP-seq or m^6^A-seq, has been widely used to globally profile m^6^A across a variety of tissues, cell types, and conditions, and it continues to be the predominant method used in most studies. Improvements to the technique have enabled single-nucleotide resolution m^6^A mapping (miCLIP and m^6^A-CLIP) (Linder et al., 2015; Ke et al., 2017), provided profiles of m^6^A within individual RNA isoforms (m^6^A-LAIC-seq) (Molinie et al., 2016), and reduced the RNA input requirements through more efficient library preparation (Zeng et al., 2018; Dierks et al., 2021).

Although widely used, antibody-based m^6^A mapping methods have their drawbacks. This includes cross-reactivity of m^6^A antibodies with m^6^A_m_, a chemically similar modification that is part of the 5′ cap structure. In addition, m^6^A site calling can be stochastic due to variability in antibody immunoprecipitation efficiency, and most studies lack sufficient replicate numbers to make accurate site calls (McIntyre et al., 2020). Furthermore, most global m^6^A mapping strategies lack the ability to quantify m^6^A stoichiometry. This has made studies of m^6^A dynamics difficult and has contributed to discrepancies in the literature regarding how m^6^A responds to cellular stress and other states.

Recently, two methods for simultaneous m^6^A mapping and quantification have overcome this problem. GLORI uses nitrous acid to deaminate unmodified A to I while leaving m^6^A unchanged. This results in unmodified A being read as G in sequencing reads, with m^6^A remaining as A (Liu et al., 2023). eTAM-seq similarly relies on exclusive deamination of unmodified A, but it does so through an evolved TadA8.20 enzyme which selectively targets unmodified A (Xiao et al., 2023). Both methods offer a simple approach for identifying m^6^A with nucleotide specificity, and they have the added advantage of being able to measure m^6^A stoichiometry transcriptome-wide. Further improvements to GLORI and eTAM-seq to limit RNA degradation will facilitate more widespread use of these methods and will help pave the way for their potential use in single-cell m^6^A mapping (below). Additionally, several other antibody-independent m^6^A profiling methods have been developed in recent years (reviewed in Owens et al., 2021). These approaches employ a variety of different strategies, including the use of methionine analogs to label m^6^A sites (m^6^A-label-seq) (Shu et al., 2020), chemical labeling of FTO-directed m^6^A demethylation intermediates (m^6^A-SEAL) (Wang et al., 2020), and treating RNA with modification-sensitive endoribonucleases (MAZTER-seq and m^6^A-REF-seq) (Garcia-Campos et al., 2019; Chen et al., 2022). Strategies for site-specific m^6^A quantification in RNAs of interest have also been developed, which serve as useful tools for investigating m^6^A within individual transcripts and/or validating the results of global m^6^A mapping for a subset of RNAs (Liu et al., 2013; Xiao et al., 2018; Castellanos-Rubio et al., 2019).

**Figure 1:**
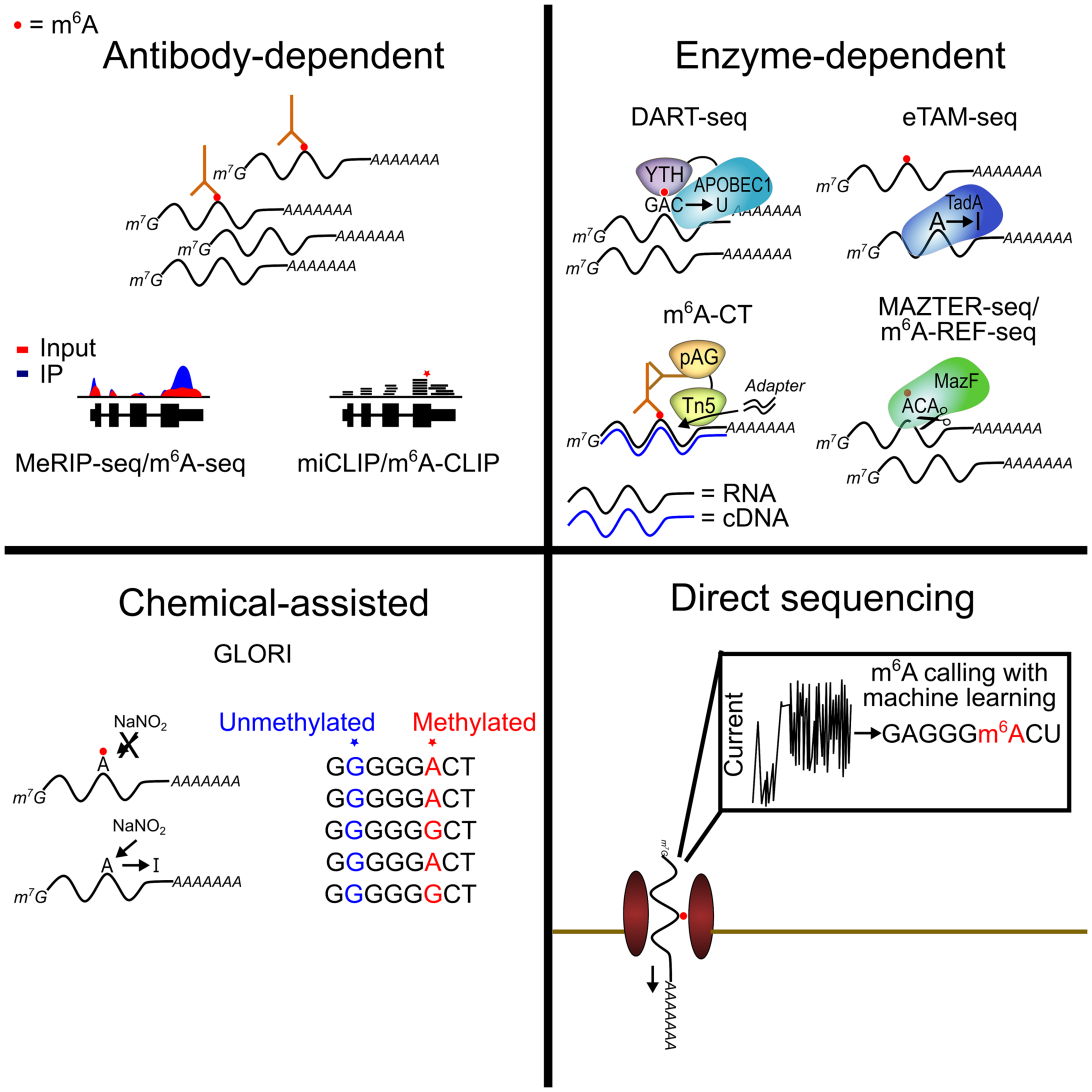
Current transcriptome-wide m^6^A profiling methods. Recent advances have provided new techniques for m^6^A profiling across the transcriptome. Some of these methods allow for highly quantitative detection of m^6^A sites at single-nucleotide resolution (GLORI and eTAM-seq). Others have facilitated the use of low-input samples, including single cells (DART-seq and m^6^A-CT).

In addition to antibody-based, enzyme-assisted, and biochemical methods for m^6^A mapping, nanopore sequencing has emerged as a technology with great promise for profiling m^6^A and other RNA modifications. This direct RNA sequencing method involves driving RNAs through a protein nanopore and measuring the variations in ionic current that occur as different nucleotides pass through the pore (Garalde et al., 2018) (Figure 1). Chemical modifications in RNAs can alter the current intensity or dwell time of the RNA as it moves through the pore, and these unique signatures can then be used to detect the presence of modifications (Jain et al., 2022). Several studies have demonstrated the ability of nanopore technology to call m^6^A sites (Zhong et al., 2023). A key advantage of this approach is that native, full-length RNA molecules can be sequenced, therefore enabling a deeper understanding of m^6^A distribution within distinct transcript isoforms, the presence of m^6^A clusters in single RNA molecules, and potential co-occurrence of m^6^A with other modifications (Leger et al., 2021; Huang et al., 2024; Mateos et al., 2024).

## Mapping m^6^A in single cells

The brain is a complex mixture of diverse cell types. However, all m^6^A profiling studies done in the brain thus far have used bulk tissue samples, which represent the cumulative m^6^A signal across different cell types and provide no information on the distribution or abundance of m^6^A within individual cells. By mapping methylated transcripts in single cells, the methylomes of all cell types can be elucidated, which would provide unprecedented insights into how m^6^A contributes to brain function and disease through influencing gene expression in distinct cell types.

Several approaches have recently been developed to achieve single-cell m^6^A profiling. Some studies have used m^6^A antibodies to perform a low-input MeRIP-seq from single cells (Li et al., 2023; Yao et al., 2023). These methods can identify methylated transcripts from individual cells, but they have some drawbacks. First, the high signal-to-noise ratio resulting from antibody enrichment complicates peak calling, especially when using low input samples. Second, these methods are generally not highly scalable and have profiled m^6^A in a few dozen cells at most (Li et al., 2023; Yao et al., 2023). The recent development of single-nucleus m6A-CUT&Tag (sn-m6A-CT) addresses these issues by coupling antibody-based methylated RNA enrichment with Tn5 transposase-mediated tagmentation (Hamashima et al., 2023). While this method still relies on m^6^A antibodies, it improves signal-to-noise relative to strategies based on immunoprecipitation alone and can be used in any cell type or tissue of interest. Furthermore, it is amenable to droplet-based library preparation methods, making it a truly high-throughput technique.

Antibody-independent strategies for single-cell m^6^A profiling have also been developed. In 2022, our group introduced single-cell DART-seq (scDART-seq), which installs a unique mutation signature adjacent to m^6^A sites (Meyer, 2019; Tegowski et al., 2022). This is achieved by expressing a fusion protein consisting of the m^6^A-binding YTH domain tethered to the cytidine deaminase APOBEC1 (Meyer, 2019). The YTH domain recruits the fusion protein to sites of methylation while APOBEC1 edits nearby cytidines to uridines, enabling m^6^A sites to be identified as C-to-T mutations in the sequencing data. This method is compatible with any scRNA-seq preparation method and does not require additional RNA processing steps, making it a highly scalable strategy which is straightforward to implement (Figure 1). However, one limitation of scDART-seq is that it requires expressing the APOBEC1-YTH protein in cells of interest. This is easy to do in many cultured cell types but can be more challenging for certain tissues. Furthermore, expression of APOBEC1-YTH could also influence cell biology if expression is prolonged (Tegowski et al., 2022).

## Emerging technologies for studying m^6^A at the single-molecule level

Most methods for m^6^A mapping rely on short read sequencing. Although these techniques can reveal m^6^A sites, they are unable to describe how these sites are distributed on individual RNA molecules. For example, many RNAs have multiple m^6^A sites, but whether these sites co-occur on the same RNA molecules is unknown. In addition, the distribution of m^6^A within distinct transcript isoforms is often difficult to assess when only a short fragment of the parent RNA is sequenced. Exploring methylation at the single-molecule level can help address these important questions.

As discussed above, nanopore sequencing has emerged as a technology with great promise for profiling m^6^A and other RNA modifications. This strategy provides information on full-length RNA molecules, which enables greater insight into the presence of modifications in splice variants or other RNA isoforms. Additionally, since RNA molecules are sequenced directly, potential biases introduced during cDNA synthesis and PCR amplification steps are avoided. However, nanopore-based RNA modification sequencing has some limitations. First, identification of modification sites requires the use of machine learning algorithms trained on datasets to enable *de novo* modification site calls, or the use of modification-free control samples to enable modification detection by comparative analysis (Hendra et al., 2022; Jain et al., 2022). For m^6^A, several computational tools have been developed for identifying methylated sites from nanopore data, with substantial variations in called sites and estimated accuracy (Zhong et al., 2023). More fundamentally, training m^6^A calling algorithms requires a known “ground-truth,” which can be difficult to know with certainty in all model systems.

In addition to DRS technologies such as nanopore sequencing, other methods exist that enable m^6^A identification in individual RNA molecules. For instance, DART-seq has been used with PacBio sequencing, which has enabled the identification of m^6^A sites along the full length of individual mRNAs (Meyer, 2019). In theory, other methods that induce m^6^A-associated mutations, such as eTAM-seq and GLORI, could also be combined with long-read sequencing to explore m^6^A on single molecules. However, these approaches have not been widely used, and given the rapid developments in nanopore technology, DRS will likely emerge as the method of choice for single-molecule m^6^A mapping.

In addition to sequencing-based approaches, other methods have been developed that enable analysis of individual methylated RNA molecules in cells. m^6^AISH-PLA uses proximity ligation between an m^6^A-recognizing antibody and a sequence-specific oligo targeted to sequences flanking the m^6^A site of interest. After ligation, rolling circle amplification (RCA) amplifies an engineered sequence recognized by a fluorescent detection probe (Ren et al., 2021). This method allows for the visualization of single methylated molecules *in situ*, facilitating novel investigations into m^6^A-mediated RNA localization and trafficking. One drawback to this method is that it cannot simultaneously visualize unmethylated transcripts, which could lead to misinterpretations if both methylated and unmethylated RNAs are similarly trafficked. However, an adaptation of the DART-seq technology, termed DART-FISH, can detect methylated and unmethylated transcripts (Sheehan et al., 2023). By expressing the APOBEC1-YTH enzyme in cells, transcripts with m^6^A-dependent C-to-U mutations can be discriminated from unmodified transcripts using padlock probe hybridization followed by RCA and hybridization of detection probes. By using distinct padlock probes for the C and U variants adjacent to an m^6^A site of interest, the unmethylated and methylated copies of an individual transcript can be visualized simultaneously. Since m^6^A has been implicated in subcellular RNA localization, approaches such as these which enable *in situ* visualization of m^6^A-modified transcripts can be powerful approaches for understanding the role of m^6^A in RNA trafficking or partitioning to subcellular compartments such as stress granules (Anders et al., 2018; Fu and Zhuang, 2020; Khong et al., 2022; Ries et al., 2023).

## Strategies for targeted m^6^A manipulation and m^6^A-dependent gene expression control

Several groups have developed tools for targeted addition or removal of m^6^A in cellular RNAs of interest. These methods involve fusing m^6^A methyltransferase or demethylase enzymes to catalytically inactive Cas proteins coupled with guide RNA (gRNA)-mediated targeting of specific transcripts. For instance, Wilson et al. fused METTL3/14 to dCas13 to achieve site-specific methylation of several cellular mRNAs, including *GAPDH*, *FOXM1*, and *SOX2*. In addition, they showed that targeted methylation of *ACTB* led to transcript degradation and that methylation of the *BRD8* and *ZNF638* transcripts impacted splicing, consistent with previous reports of m^6^A function in these mRNAs (Wilson et al., 2020). Li et al. showed that ALKBH5 tethered to dCas13b can remove m^6^A from oncogenic transcripts *EGFR* and *MYC* in the presence of transcript-targeting gRNAs, leading to decreased protein expression and reduced cell proliferation (Li et al., 2020). Tethering of dCas9 to m^6^A methyltransferases and demethylases has also been used to achieve targeted m^6^A writing and erasing, respectively (Liu et al., 2019). Collectively, these tools have utility not only for basic research into m^6^A function but also as a potential therapeutic strategy to overcome the effects of hyper or hypomethylation during disease. Current challenges include minimizing off-targeting effects to ensure transcript specificity and optimization of methylation and demethylation efficiency. However, the use of CRISPR/Cas-based technologies for targeting RNA has accelerated at a rapid pace, and as these and other methods continue to expand, we anticipate that the tools for manipulating m^6^A and other RNA modifications will also improve. Indeed, these methods have already been expanded to include light-activated m^6^A modification systems which add temporal specificity (Lan et al., 2021; Shi et al., 2022).

The tools above use targeted manipulation of m^6^A levels in specific RNAs to control the expression of genes of interest. This holds promise as a potential therapeutic strategy, since m^6^A dysregulation can lead to abnormal expression of specific genes to promote the pathogenesis of cancer and other diseases (Yang et al., 2020; Jiang et al., 2021; Delaunay et al., 2024). However, an alternative approach is to couple the presence of m^6^A with the expression of desired proteins. Recently, our group developed a genetically encoded m^6^A sensor system (GEMS), which couples mRNA methylation with expression of a protein of interest (Marayati et al., 2024). This is achieved by expressing a reporter mRNA together with APOBEC1-YTH in cells. The reporter mRNA contains an m^6^A sensor sequence that, when methylated, recruits APOBEC1-YTH to convert nearby cytidines to uridines, in turn generating one or more stop codons that block translation of a degradation tag after the coding sequence of the protein of interest. The result is stable protein production only when the mRNA is methylated. We used this system to achieve m^6^A-coupled expression of tumor suppressor proteins in cancer cells, which led to decreased cell proliferation and migration (Marayati et al., 2024). Although m^6^A-coupled protein expression technologies such as this still require further optimization, the ability to sense m^6^A in living cells offers an attractive platform both for methylation-sensitive protein expression as well as for studies of m^6^A dynamics in the brain and other tissues.

## Discussion

Much of our understanding of m^6^A in the brain has been driven by recent advances in m^6^A mapping technologies. These tools have not only enabled the identification of methylated RNAs within the brain and other tissues but have also provided a deeper understanding of m^6^A dynamics and function. Although antibody-based methods have been the predominant method of choice for transcriptome-wide m^6^A mapping, newer approaches have emerged in the last few years which overcome many of the limitations of antibody-based approaches. For instance, GLORI and eTAM-seq offer not only nucleotide-resolution m^6^A mapping, but they also enable quantification of m^6^A stoichiometry. The ability to measure changes in m^6^A abundance is an important advance, since methods for reliable, sensitive quantification of m^6^A stoichiometry transcriptome-wide have been largely elusive, which has contributed to discrepancies regarding the dynamic nature of m^6^A. Although GLORI and eTAM-seq have great potential for becoming the new gold standard of m^6^A mapping and quantification, further refinements of these methods to improve sensitivity and reduce RNA degradation will be needed for their widespread adoption.

Direct RNA sequencing with nanopore technology also holds great promise for enabling m^6^A identification at the single-molecule level and within different transcript isoforms. Additionally, nanopore sequencing can potentially be used to identify multiple modifications within a single RNA molecule, which is an area that we currently have little knowledge about. However, achieving these goals will require improved throughput and accuracy, as well as establishment of consistent data analysis pipelines and appropriate training datasets. Nevertheless, rapid progress is being made in nanopore-based modification mapping, so we anticipate that this technology will become increasingly widespread in the coming years.

The ability to map m^6^A in single cells is an important step forward for deepening our understanding of m^6^A regulation and function. The recent development of scDART-seq (Tegowski et al., 2022), scm^6^A-seq (Yao et al., 2023), picoMeRIP-seq (Li et al., 2023), and single-nucleus m^6^A-CUT&Tag (sn-m^6^A-CT) (Hamashima et al., 2023) have been critical advances and have revealed new insights into m^6^A distribution and regulation within individual cells of a population. Applying single-cell m^6^A mapping methods to the brain will undoubtedly uncover new information about m^6^A dynamics and regulation within distinct brain cell types. In particular, our understanding of m^6^A function in non-neuronal cells is limited, so such studies will greatly facilitate future discoveries in this area.

Going forward, it will be important to further develop single-cell m^6^A mapping technologies to enable their widespread use across cell or tissue types of interest. Additionally, methods such as GLORI or eTAM-seq may be promising antibody-independent strategies for single-cell m^6^A mapping, but their sensitivity for low-input RNA must be further developed, and their propensity to induce RNA degradation must be addressed. Nevertheless, this is an exciting time for m^6^A mapping technology in single cells, with a few tools already available and further developments undoubtedly on the horizon. Having the ability to combine m^6^A mapping with other single-cell “omics” technologies will be very powerful for furthering our understanding of the interplay between m^6^A and other gene regulatory processes such as chromatin remodeling, transcription regulation, and RNA processing events.

In addition to technologies for mapping and quantifying m^6^A, there are emerging tools for targeted manipulation of m^6^A which can achieve selective methylation or demethylation of RNAs of interest. The ability to selectively add or remove m^6^A from RNAs is a useful tool for investigating m^6^A function. However, one consideration is that m^6^A sites cluster in RNAs, and recent studies have indicated that cellular RNAs contain many more m^6^A sites than previously thought (Tegowski et al., 2022; Liu et al., 2023). Thus, the effects of adding or removing a single m^6^A site may be compensated for through methylation of other nearby adenosines within a given region of methylation. This is also an important consideration when developing m^6^A targeting tools for therapeutic applications, as multiple m^6^A sites may exist at nearby positions in a transcript of interest. However, methylating or demethylating single sites has been shown to impact RNA expression in cells (Liu et al., 2019; Li et al., 2020; Wilson et al., 2020), suggesting that compensation by nearby m^6^A sites does not happen for all RNAs. It is also possible that the individual m^6^A sites that make up methylation “clusters” occur on different RNA molecules, which would make compensation by nearby adenosines less likely. Most m^6^A profiling strategies do not report the individual RNA molecules in which m^6^A sites reside, underscoring the need to develop better tools for single-molecule m^6^A mapping.

Going forward, it will be important for the field to address issues related to sensitivity and reproducibility of methods for studying m^6^A. Newer technologies such as GLORI and eTAM-seq that enable high-resolution m^6^A mapping as well as quantification can potentially enable better insights into m^6^A dynamics, since many m^6^A sites may be regulated by changes in abundance as opposed to strict gain or loss of methylation. Additionally, the recent development of tools for sensing m^6^A provide new opportunities for studying m^6^A dynamics in living cells, in contrast to other methods that require RNA isolation. Our understanding of how m^6^A is regulated within the brain during both healthy and disease states will undoubtedly be accelerated by the ability to map and quantify m^6^A within the brain and in specific cell types. Thus, further development of single-cell m^6^A profiling approaches will be important. Finally, nanopore sequencing or other methods that provide single-molecule information have the potential to provide deeper insights into roles of m^6^A in distinct transcript isoforms, as well as the possibility of multiple different modifications co-occurring on the same RNAs. We anticipate that continued development of these methods in the coming years will make them more widely used for studies of m^6^A in the brain.

## Data availability statement

The original contributions presented in the study are included in the article/supplementary material, further inquiries can be directed to the corresponding author.

## Author contributions

MT: Writing – original draft, Writing – review & editing. KM: Writing – original draft, Writing – review & editing.
